# Optimization Based Tumor Classification from Microarray Gene Expression Data

**DOI:** 10.1371/journal.pone.0014579

**Published:** 2011-02-04

**Authors:** Onur Dagliyan, Fadime Uney-Yuksektepe, I. Halil Kavakli, Metin Turkay

**Affiliations:** 1 Department of Chemical and Biological Engineering, Koc University, Istanbul, Turkey; 2 Department of Industrial Engineering, Istanbul Kultur University, Istanbul, Turkey; 3 Department of Industrial Engineering, Koc University, Istanbul, Turkey; Centro de Investigación Príncipe Felipe, Spain

## Abstract

**Background:**

An important use of data obtained from microarray measurements is the classification of tumor types with respect to genes that are either up or down regulated in specific cancer types. A number of algorithms have been proposed to obtain such classifications. These algorithms usually require parameter optimization to obtain accurate results depending on the type of data. Additionally, it is highly critical to find an optimal set of markers among those up or down regulated genes that can be clinically utilized to build assays for the diagnosis or to follow progression of specific cancer types. In this paper, we employ a mixed integer programming based classification algorithm named hyper-box enclosure method (HBE) for the classification of some cancer types with a minimal set of predictor genes. This optimization based method which is a user friendly and efficient classifier may allow the clinicians to diagnose and follow progression of certain cancer types.

**Methodology/Principal Findings:**

We apply HBE algorithm to some well known data sets such as leukemia, prostate cancer, diffuse large B-cell lymphoma (DLBCL), small round blue cell tumors (SRBCT) to find some predictor genes that can be utilized for diagnosis and prognosis in a robust manner with a high accuracy. Our approach does not require any modification or parameter optimization for each data set. Additionally, information gain attribute evaluator, relief attribute evaluator and correlation-based feature selection methods are employed for the gene selection. The results are compared with those from other studies and biological roles of selected genes in corresponding cancer type are described.

**Conclusions/Significance:**

The performance of our algorithm overall was better than the other algorithms reported in the literature and classifiers found in WEKA data-mining package. Since it does not require a parameter optimization and it performs consistently very high prediction rate on different type of data sets, HBE method is an effective and consistent tool for cancer type prediction with a small number of gene markers.

## Introduction

Microarray technology provides wealth of information on expression levels of thousand genes that has been used for diagnostic and prognostic purposes for various types of diseases. The data obtained from microarray measurements leads to understanding of genes that are being regulated under the disease conditions including cancer both in biology and clinical medicine at the molecular level [1]. Cancer is the most deadly genetic disease, and it occurs either through acquired mutations or epigenetic changes that lead to altered gene expressions profile of cancerous cells. Consequently, microarray technology is employed to identify up or down regulated genes that play a role on the specific cancers, activation of oncogenic pathways, and to discover novel biomarkers for the clinical diagnosis [2]. However, such approach is an expensive, time-consuming process, and not practical in terms of clinical application for each patient. Researchers cannot effectively benefit from the current microarray technology completely due to limitations of the algorithms being used for data analysis. Building a set of marker genes with data classification enable to assess the progression cancer. The number of genes (features) considered in the analysis of microarray data is very critical. A very small number of genes usually cannot yield reliable results, whereas very large number of genes decreases the information by adding noise [3]. Therefore, it is necessary to find an optimal set of genes for each cancer type as predictors that help to classify different labeled cells with high prediction accuracy.

An important characteristics of microarray data is the large number of genes relative to number of samples. This high dimensionality in gene space increases the computational complexity while it usually decreases the accuracy of the classification. This fact brings the necessity of gene selection by ranking or gene reduction for the high dimensional gene space. The relevance of genes in cancer occurrence can be categorized into three classes: Strongly relevant, weakly relevant and irrelevant genes [4]. Strongly relevant genes are the ones that have been shown in cancer cell formation and always needed in the optimal set, whereas the weak relevant genes are necessary for the optimal set at some conditions. Therefore it is important to select genes that are used for the identification of diseases for the following reasons: 1) making the classification easier by revealing only the relevant genes 2) improving the classification accuracy 3) reducing the dimensionality of the data set [5]. In an effort to choose the optimal subset of predictor genes, different methods such as neighborhood analysis [6], bayesian variable selection [7], principle component analysis [8], genetic evolution of subsets of expressed sequences (GESSES) [9] are employed.

The effectiveness of the selected gene subset is measured by its prediction accuracy or error rate in classification. IN microarray experiments, classification of data is a crucial step for the prediction of phenotype of cells. Different machine learning approaches have been employed to analyze microarray data including k-nearest-neighbors [6], [10], artificial neural networks [8], support vector machines [11]–[13], maximal margin linear programming [14], and random forest [15]. However, all of these algorithms require parameter optimization depending on the structure of data set. For example, two different parameters must be used in classification of two different cancer types; therefore, the optimal parameter values must be found for each data set. Our approach based on mixed integer programming is highly effective in different applications such as protein fold type prediction [16] and drug classification [17]–[19] without requiring any parameter optimization.

In this work, we show that a systematic and efficient algorithm, mixed integer linear programming based hyper-box enclosure (HBE) approach, can be applied to classification of different cancer types efficiently. We first introduce and establish a consistent classification method for different types of microarray data. Second we provide an optimal set of genes as best diagnostic indicators for different cancer types that gives the highest accuracy in classification. Information gain attribute evaluator, relief attribute evaluator and correlation-based feature selection (CFS) methods are used for gene selection. We conduct experiments using 6 well known cancer data sets including leukemia data set [6], two prostate cancer data sets [20], lymphoma [21], diffuse large B-cell lymphoma (DLBCL) [22], small round blue cell tumors (SRBCT) [8]. Moreover, biological interpretation of selected genes is presented with the explanation of their relationship to the related cancer types from the experimental results available in literature.

## Results and Discussion

We have selected the most widely used data sets in the literature for the evaluation of our algorithm. These data sets were obtained from Keng Ridge Bio-Medical (http://datam.i2r.a-star.edu.sg/datasets/krbd/) and Artificial Intelligence Laboratory (http://www.ailab.si/supp/bi-cancer/projections/index.htm) databases (Table 1). All data sets and scripts to run the proposed classification approach are provided as Supporting Information S2.

**Table 1:** Cancer data sets used in this study.

Data set	Samples	Genes	Classes	Reference
Leukemia	72	7129	2	Golub *et al*. (1999)
Prostate cancer	102	12600	2	Singh *et al*. (2002)
Prostate outcome	21	12600	2	Singh *et al*. (2002)
DLBCL	77	7129	2	Shipp *et al*. (2002)
Lymphoma	47	4026	2	Alizadeh *et al*. (2000)
SRBCT	83	2308	4	Khan *et al*. (2001)

### Leukemia, AML-ALL

This data [6] consist of two types of leukemia, acute lymphoblastic leukemia (ALL), and acute myeloid leukemia (AML). Each sample was obtained from bone marrow and was analyzed using Affymetrix microarrays containing 7129 genes. The training data consists of 38 samples (27 ALL and 11 AML), and the test data consists of 34 samples (20 ALL and 14 AML). Table 2 shows the classification accuracies of different algorithms on this data set. The HBE method classifies all test samples perfectly, and gives the best leave-one-out (LOOCV) result (98.61%) together with the logistic regression classifier. RBF Network and HBE method gives average of 97.43% and 97.14% accuracy with ten-fold cross validation (10-CV) respectively. As an additional evaluation, HBE method classifies all test samples perfectly using 4 genes proposed by Golub *et al*. [6] and gives a classification accuracy of 98.57% using ten-fold cross validation. Tan and Gilbert [23] report 91.18% (10-CV) with 1038 genes using Bagging and AdaBoost methods. Dettling and Buhlmann [24] obtained the accuracy of 98.61% (LOOCV) with 3 gene clusters using aggregated trees method, where gene clusters are reported as a minimum number of 1 and maximum number of 23. Nguyen and Rocke [25] correctly classifies 33 out of 34 with 50 genes using partial least squares (PLS) and Logistic Discrimination (LD) classification. Lee *et al*. [26] misclassifies one sample in test using 5 genes. Deutsch [9] uses an iterative algorithm to obtain high classification accuracy with minimum number of genes. Test samples are classified with an average dimension of 9. Antonov *et al*. [14] could predict all samples in test set and obtained the accuracy of 98% (LOOCV) using 132 genes. Chen *et al*. [27], obtained perfect test set accuracy with minimum 7 genes. Consideration of all these results shows that HBE method is the most accurate classifier on leukemia data set considering all validation methods including test set validation, ten-fold cross validation and leave-one-out validation.

**Table 2:** Classification results of leukemia (AML-ALL) data set.

Classifier	Test Set	10-CV	LOOCV
HBE	**100**	97.14±0.903	**98.61**
BayesNet	94.12	95.71	95.83
LibSVM	58.82	86.57±10.44	91.67
SMO	97.06	93.14±0.571	94.44
Logistic Regression	91.18	96.86±1.67	**98.61**
RBF Network	97.06	**97.43±1.07**	97.22
IBk	97.06	96.00±1.40	95.83
J48	94.12	89.14±1.94	90.28
Random Forest	94.12	93.14±1.07	90.2

All the genes that have been selected by HBE method are also selected by Lee *et al* in their significant gene pool that consists of 27 genes that include redundant genes. Table 3 shows the genes overlapping with the selected genes by other groups. The highest accuracy is obtained with the optimal gene set consisting of 4 genes: Myeloperoxidase (M19507-at), adipsin (M84526-at), CD33 antigen and TCF3 transcription factor 3. In fact, previous experimental and clinical studies indicated that these genes are associated with Leukemia. Myeloperoxidase is a peroxidase enzyme that produces hypochlorous acid from hydrogen peroxide and chloride anion. In recent years, myeloperoxidase staining is used in the diagnosis of acute myeloid leukemia to show that the leukemic cells were obtained from the myeloid lineage [28]. Our results are in agreement with Chen *et al*. [27] where myeloperoxidase was also selected. Additionally, Chu *et al*. [29] also selects MPO myeloperoxide. The membrane antigen CD33 is a sialic acid-dependent cell adhesion molecule is a membrane protein. CD33 is highly expressed on the surface of leukaemic blasts. About 85%–90% of acute AML cases are considered to be CD33 positive [30]. CD33 is constitutively expressed in haematopoietic progenitors, but at significantly lower membrane density than in leukaemia cells [31]. Therefore, CD33 represents an interesting target for antibody-based anti-leukaemic therapies. This gene is also selected as an important gene in significant gene subset studies conducted by other researchers [29], [32], [33]. Transcription factor 3 (TCF3) plays an important role with tissue-specific basic helix-loop-helix (bHLH) in embryogenesis [34]. The TCF3-HLF fusion transcription factor generated by t(17;19)(q22;p13) translocation is found in a small subset of pro-B cell acute ALL and promotes leukemogenesis by substituting for the antiapoptotic function of cytokines [35]. Also it has been shown that TCF3 level is up-regulated in ALL patients due to this translocation. Additionally, this protein is the cause of forms of pre-B-cell acute lymphoblastic leukemia [36]. Also, [29] *et al*. also selects this gene as one of the biomarkers in 14 significant gene subset. Although adipsine is a serine protease homolog which is synthesized and secreted by adipose cells and is found in the bloodstream has been shown to plays a role in myeloid cell differentiation [37]. Sakhinia *et al*. [38] indicated that gene expression is up-regulated in acute AML patients by real time PCR. Additionally [29] *et al*. also selects this gene as a biomarker.

**Table 3:** Selected genes which overlap with genes selected by other groups (Leukemia data set).

Gene	Reference
Myeloperoxide	[27], [26], [67], [14]
CD33	[26], [67], [5], [32], [6], [61]
TCF3	[26], [67], [61], [68]
Adipsin	[26], [67], [61], [68]

### Prostate Cancer

The prostate cancer data consists of 102 tissue samples (52 prostate tumor and 50 normal tissues) with 12,600 genes. Considering the results given in Table 4, HBE method is the most accurate classifier among others with LOOCV (96.08% accuracy) and it is the second most accurate classifier with Bayesnet at an average of 94.80% accuracy with ten-fold cross validation (10-CV). The support vector machines method SMO reports an average of 95.20% accuracy with 10-CV. Tan and Gilbert [23] report 75.53% (10-CV) with 2071 genes using Bagging method. Hewett and Kijsanayothin [33] obtain the accuracy of 91.18% (10-CV) with 6 genes using SVM on prostate cancer data set. Statnikov *et al*. [11] obtain 92% (10-CV) accuracy without any gene selection. Dettling and Buhlmann [24] report 95.10% (LOOCV) with 3 gene clusters (clusters consist of minimum 1 gene and maximum 17 genes) using nearest neighbor method. Similarly, Fort and Lambert-Lacroix [39] get 95.10% (LOOCV) with 1000 genes using Ridge PLS method. Xiong and Chen [40] choose 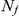 most discriminatory genes where 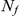 takes values between 10 and 2000. They repeated the experiment 100 times for each 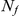 value and obtained an average value of 94.78% using uncorrelated linear discriminant analysis. Finally, Zhang and Deng [41] report an accuracy of 96.08% (LOOCV) using SVM with 13 genes.

**Table 4:** Classification results of prostate cancer data set.

Classifier	10-CV	LOOCV
HBE	94.80±0.4	**96.08**
BayesNet	94.80±1.17	95.10
LibSVM	94.60±1.36	95.10
SMO	**95.20±0.4**	95.10
Logistic Regression	90.00±1.10	92.16
RBF Network	93.20±0.75	93.14
IBk	93.40±1.74	93.14
J48	88.00±1.095	90.20
Random Forest	92.60±0.49	94.12

The selected genes by HBE are serine protease hepsin (X07732), nel-related protein 2 (D83018), ao89h09.x1 (AI207842), Cdk-inhibitor p57KIP2 (U22398), DKFZp564I1663-r1 (AL036744), adipsin/complement factor D (M84526), glutathione transferase 4 (GSTM4) (M96233), DKFZp586K1220 (AL050152), aldose reductase (X15414), ADP/ATP translocase (J03592). In fact, many of the genes that have been selected in this study shown to display different expression patterns in prostate cancer tissues. For example hepsin (a cell surface serine protease) is significantly upregulated in human prostate cancer and it promotes cancer progression and metastasis of prostate [42]. It is reported that the expression of p57Kip2 is dramatically decreased in human prostate cancer and the overexpression of p57Kip highly suppresses the cell proliferation [43]. Furthermore, another selected gene glutathione transferase mediates the proliferation of androgen-independent prostate cancer cells [44]. Finally, it has been shown that aldose reductase gene is responsible in carbohydrate metabolism that converts glucose to sorbitol [45].

### Prostate Cancer Outcome

This data contains 8 patients having relapsed and 13 patients having non-relapse with measurements of 12600 genes.

Table 5 summarizes the classification results for the of prostate cancer outcome data set. We performed leave-one-out-validation only since there are 21 samples in the data. As it is seen in Table 5, HBE method is one of the top classifiers together with BayesNet and RBF Network with the accuracy of 95.24% compared with other methods. Tan and Gilbert [23] report 85.71% (10-CV) using Bagging method with 208 genes. Comparing to the results of Tan and Gilbert [23], HBE method gives an accuracy of 90% with ten-fold cross validation using only three genes.

**Table 5:** Classification results of prostate cancer outcome data set.

Classifier	LOOCV
HBE	**95.24**
BayesNet	**95.24**
LibSVM	61.90
SMO	57.14
Logistic Regression	47.62
RBF Network	**95.24**
IBk	80.95
J48	85.71
Random Forest	90.48

The genes, selected with HBE are shown to play a critical role in prostate cancers, are human cofactor A protein (AF038952), farnesyl-protein transferase beta-subunit (HUMFPTB), glutamine-fructose-6-phosphate amidotransferase (GFAT) (M90516). The function of heterodimeric enzyme farnesyl: protein transferase (FPTase) 3 is to transfer of a 15-carbon isoprenoid moiety to a C-terminal cysteine of many cellular proteins. The inhibition of farnesyl protein transferase affects for the prevention of proper functioning of the Ras protein that leads to oncogenesis or cancer. In fact, elimination of cancer were reported to increase when prostate cancer cell line is treated with farnesyl-protein transferase inhibitor [46]. Glutamine:fructose-6-phosphate amidotransferase (GFA), the first and rate-limiting enzyme in the hexosamine biosynthetic pathway, transfers the amide group from glutamine to fructose-6-phosphate to form glucosamine-6-phosphate (GlcN-6-P), a precursor of uridine diphosphate-N-acetyl-glucosamine. It has been demonstrated that overexpression of GFA in Rat-1 fibroblasts causes insulin resistance [47].

### The diffuse large B-cell lymphoma (DLBCL)

This data set contains 58 samples from DLBCL patients and 19 samples from follicular lymphoma. The gene expression profiles were analyzed using Affymetrix human 6800 oligonucleotide arrays. HBE method is the most accurate classifier among the classifiers with both 10-CV and LOOCV in the DLBCL data set (Table 6). From the literature, GESSES method predicts all samples correctly with different random numbers of starting top genes (from 77 to 130) [9]. The final predictors ranged in number of genes, from four to twelve. Statnikov *et al*. [11] reaches the accuracy of 97.50 (10-CV) without gene selection. Zhang and Deng (2007) report a classification accuracy of 92.71% (LOOCV) using kNN (k = 5) with 8 genes.

**Table 6:** Classification results of DLBCL.

Classifier	10-CV	LOOCV
HBE	**92.25±1.46**	**96.10**
BayesNet	89.0±0.94	89.61
LibSVM	83.5±0.94	84.42
SMO	88.25±1.0	89.61
Logistic Regression	87.75±1.46	89.61
RBF Network	90.5±1.5	93.51
IBk	87.5±0.79	88.31
J48	88.25±1	89.61
Random Forest	89.75±2.15	89.61

HBE analysis resulted in high classification accuracy with 6 genes which are DNA replication licensing factor CDC47 homolog (D55716-at), gamma-interferon-inducible protein IP-30 precursor (J03909-at), LDHA lactate dehydrogenase A (X02152-at), CD69 antigen (Z30426-at), SLC (AB002409-at), Rad2 (HG4074-HT4344-at). When the function of those proteins and their role in DLBCL were analyzed, we see that these proteins play a significant role in the progression of DLBCL. DNA replication licensing factor CDC47 is a factor that helps the DNA to undergo a single round of replication per cell cycle. CDC47 is not only necessary for DNA replication and cell proliferation, but also for S-phase checkpoint activation upon UV-induced damage [48]. The function of LDHA lactate dehydrogenase is to catalyze the conversion of L-lactate and NAD to pyruvate and NADH in the last step of anaerobic glycolysis. It is proved that mutations in lactate dehydrogenase A gene causes the exertional myoglobinuria [49]. CD69, known as early T cell activation antigen, is expressed on a variety of immune cells, including T- and B- lymphocytes, NK cells, monocytes/macrophages, and granulocytes [50]. Secondary lymphoid-tissue chemokine (SLC) is a cytokine belonging to the CC chemokine family and it is constitutively expressed in a variety of lymphoid tissues. This gene is a potent and specific chemoattractant for lymphocytes [51]. Rad 2 (Flap endonuclease 1) is a member of the XPG/RAD2 endonuclease family and is involved in DNA repair [52].

### Lymphoma

The lymphoma data set contains 47 samples, 24 of them are referred to as germinal center B-like group and 23 are activated B-like group. This data set contains expression of 4026 genes.

In the lymphoma data set, HBE method is the most accurate classifier using both ten-fold-cross-validation and leave-one-out cross validation with the accuracies of 96.40% and 97.87% respectively (Table 7). Support vector machine algorithm, SMO, is the second highest accurate classifier with the accuracy of 96.00% with both of the validation methods. Hewett and Kijsanayothin [33] have obtained a classification accuracy of 97.87% (10-CV) with SVM and Bayesian network methods. Dettling and Buhlmann [24] reports an the accuracy of 100% (LOOCV) with 10 gene clusters (min: 1 and max: 16 of genes) using nearest neighbor method. The best accuracy reported is in the literature including this presented paper, Zhang and Deng [41] with 100% accuracy (LOOCV) using SVM with 3 genes. However, the specific information of those genes were not reported.

**Table 7:** Classification results of Lymphoma.

Classifier	10-CV	LOOCV
HBE	**96.40±1.5**	**97.87**
BayesNet	95.20±0.98	93.62
LibSVM	94.4±0.8	93.62
SMO	96.00	95.75
Logistic Regression	92.4±2.65	91.49
RBF Network	95.20±0.98	95.75
IBk	95.2±1.6	95.75
J48	81.6±2.33	87.23
Random Forest	92.00±1.79	89.36

algorithm selects the following genes: Deoxycytidylate deaminase (19408), lymphoid-restricted membrane protein (JAW1) (16886), PKU-beta = KIAA0137 = protein kinase (20423), T-cell protein-tyrosine phosphatase (17140), TTG-2 Rhombotin-2 (19238), stress-activated protein kinase (JNK3) (19384), and unknown labeled genes with ids 19288, 19274, 13394. Expression of the following genes are currently being used as markers for lymphoma in clinical diagnosis. Deoxycytidylate deaminase (dCMPase) hydrolyzes dCMP into dUMP, and it is suggested that this gene is a marker of the aggression of human lymphoid malignancies [53]. Jaw1, also known as lymphoid-restricted membrane protein (LRMP), is an endoplasmic reticulum-associated protein. It is known that the expression of Jaw1/LRMP mRNA is high in germinal center B-cells and in diffuse large B-cell lymphomas of ‘germinal center’ subtype [54]. In addition, the following genes were selected and their expression patterns were reported to be greatly changed in lymphoma. Among these genes, PKU-beta, a serine/threonine protein kinase, has role in chromatin remodeling, DNA replication and mitosis [55]. T-cell protein tyrosine phosphatases, phospho tyrosine-specific protein phosphatase, nuclear dephosphorylation of phospho-STAT6 (pSTAT6) was observed in activated-B-cell (ABC)-like tumors. Moreover, TTG-2 Rhombotin-2 is a cysteine rich protein with LIM motif and immunohistologic analysis show that LMO2 protein is expressed as a nuclear marker in normal germinal-center (GC) B cells and GC-derived B-cell lines and in a subset of GC-derived B-cell lymphomas [56]. Finally, stress-activated protein Jun N-terminal kinase (JNK3) is a member of mitogen-activated protein kinase (MAPK) superfamily and it plays an important role in signaling pathways of critical physiological processes, including apoptosis, differentiation and proliferation. It is known that the activation of JNK leads to the interferon-alpha-induced apoptosis in B-cell lymphoma [57].

### Small round blue cell tumors

There are four different small round blue cell tumors in this data set: Ewing family tumor (EWS), Burkitt lymphoma (BL), neuroblastoma (NB) and rhabdomysarcoma (RMS). The training set contains 63 samples and the test set contains 20 samples. The cDNA microarrays comprise 2308 genes. Small round blue cell tumors (SRBCT) of childhood are diagnosed using single layer neural network [8] where the number of genes in the data set was reduced to 96 to predict the classes of the test data perfectly.

Table 8 shows that HBE method outperforms other classifiers using all validation methods. It gives perfect classification on the test set with 5 genes. Moreover, it has an average of 97.5% of accuracy using ten-fold-cross validation and 96.39% with leave-one-out-cross validation. Comparing to other studies in the literature, Dettling and Buhlmann [24] has obtained 100% (LOOCV) with 1 gene cluster (minimum: 1 gene maximum: 14 genes) using nearest neighbor method. Deutsch [9] predicts all test samples when 100 predictors were used, where the average number of genes in a predictor was 12.7. Statnikov *et al*. [11] obtain perfect accuracy using ten-fold-cross validation with many methods without gene selection. Finally, Chen *et al*. [52] perfectly classifies all samples in the test set using 10 genes with SVM and kernel Fisher discriminant analysis. Considering these studies, HBE method is the most robust method, since it has highest accuracy with the least number of genes on not only test set but also using other types of validations including ten-fold and leave-one-out cross validation.

**Table 8:** Classification results of SRBCT.

Classifier	Test Set	10-CV	LOOCV
HBE	**100**	**97.5±2.5**	**96.39**
BayesNet	85	94.5±1.5	95.18
LibSVM	90	84.75±0.94	84.34
SMO	95	89.5±3.22	93,98
Logistic Regression	80	91.5±1.66	91.57
RBF Network	90	93.25±1.0	93.98
IBk	90	92.25±0.5	92.77
J48	90	88.75±0.79	91.57
Random Forest	95	89.75±1.66	92.77

The selected genes with their gene ids in SRBCT classification are Fc fragment of IgG, receptor, transporter, alpha (FCGRT) (70394), transmembrane protein (812105), fibroblast growth factor receptor 4 (784224), ESTs (295985), recoverin (383188). FCGRT encodes a receptor binding the Fc region of monomeric immunoglobulin G. This protein both helps to transfer of immunoglobulin G antibodies from mother to fetus across the placenta, and binds to immunoglobulin G to prevent the antibody degradation [58]. Growth factor receptors (FGFRs) bind fibroblast growth factors which play key roles in proliferation and differentiation of different type of cells and tissues [59]. Recoverin is neuronal calcium-binding protein that plays a role in the inhibition of rhodopsinosopsin kinase which is a regulator in the phosphorylation of rhosopsin [60]. Zhoua *et al*. [61] also select FCGRT, transmembrane protein, ESTs, recoverin in their significant gene pool (Table 9). Chen *et al*. [27] selects FCGRT and fibroblast growth factor receptor 4.

**Table 9:** Selected genes which overlap with genes selected by other groups (SRBCT data set).

Gene	Reference
FCGRT	[27], [61]
Transmembrane protein	[61]
Fibroblast growth factor receptor	[27]
ESTs	[61]
Recoverin	[61]

### Conclusion

The contributions of this work are two-fold. The first contribution is that we implement an effective optimization based classifier that gives very high performance and valuable insight into different type of cancer data sets. Previously it has been shown that our approach was successfully applied to protein folding and drug classification problems. HBE approach does not require parameters to optimize in order to obtain high classification accuracies. This method can be used for different types of data without any modifications. The second contribution is finding of optimal predictor genes that give the highest accuracy in classification. This effort can provide to develop antibody assays for the diagnosis of specific types of cancer and to provide accurate diagnostics by only measuring expression of few genes. We have applied our algorithm on publicly available data sets including leukemia data set, two prostate cancer data sets, two lymphoma data sets and SRBCT data set. In conclusion, mixed-integer programming based hyper-box enclosure approach is robust and effective method for microarray analysis.

## Methods

### Preprocessing and Gene Selection

We performed scaling to all data sets by using the expression of
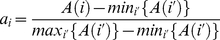
(1)where 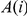 is the gene expression value of 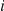
th sample and 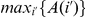 and 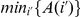 are the maximum and minimum gene expression levels respectively. Additionally, in cross validation runs, the data was randomly divided into 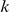-fold.

There are generally three types of approaches in feature (gene) selection: Filters, wrappers, and feature weighting. Filter methods eliminate irrelevant features according to some prior knowledge. Wrapper approaches use machine learning algorithms to evaluate the feature subsets; however, they have high computational complexity when they combined with classification algorithms. Feature weighting methods simply weigh features instead of selecting a subset of features that is a combinatorial problem. We employed information gain attribute evaluator, relief attribute evaluator, and correlation-based feature selection (CFS) from Weka machine learning package [62] for the gene selection. The details of these algorithms can be found in the work of Wang *et al*. [5].

Information gain evaluates a feature (gene) by measuring the information gain with respect to the class:
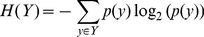
(2)where 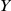 and 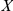 are the features, 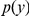 is the marginal probability density function for random variable 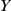. Equation 1 provides the entropy of 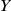. Entropy is a measure of uncertainty in information theory. There is a relationship between feature 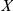 and 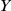 when following cases are ensured: i) expression values of feature 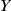 in the training set are partitioned in due to the expression values of second feature 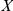 ii) the entropy of 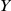 prior to partitioning is higher than the entropy of 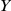 with respect to the partitions induced by 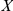. The entropy of 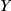 after observing 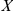 is given in Equation 2.
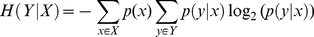
(3)

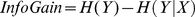
(4)where 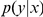 is the conditional probability of 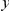 given 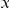. Information Gain (Equation 3) is a measure of additional information about 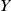 provided by 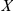 representing the amount by which the entropy of 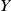 decreases.

Relief attribute evaluator is an evaluating algorithm that rates features (genes in our case) due to these facts: (1) how well their values distinguish among samples of different classes (tumor type in our case) (2) how well they cluster instances of the same class [63].

Correlation-based feature selection (CFS) is a fast algorithm that reveals a good feature subset that contains features highly correlated with the class, yet uncorrelated with each other [64].
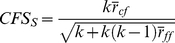
(5)where 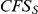 is the score of a feature subset 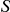 containing 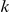 features, 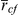 is the average feature to class correlation 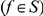, and 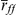 is the average feature to feature correlation. The numerator of Equation 4 indicates how predictive of the class a group of features are and the denominator is a measure of redundancy among that group of features.

### Classification: Hyper-box enclosure algorithm

The objective in data classification is to assign samples that are described by several attributes into a predefined number of classes. In this study, we propose HBE algorithm for the classification of cancer types. This algorithm consists of integer programming (IP) and mixed integer linear programming (MILP) based components, and the data points belonging to different classes are discriminated by hyper-boxes. The use of hyper-boxes for defining boundaries of the sets that include all or some of the samples in that set can be very accurate on both two-class (normal/cancer) and multi-class (more than two tumor types) problems. If it is necessary, more than one hyper-box could be used in order to represent a class. When two classes are both represented with a single hyper-box respectively, the boundaries of these hyper-boxes may overlap. Thus, two boxes could be constructed in order to eliminate this overlapping. The description of the optimization model is given in Supporting Information S1.

**Figure 1:**
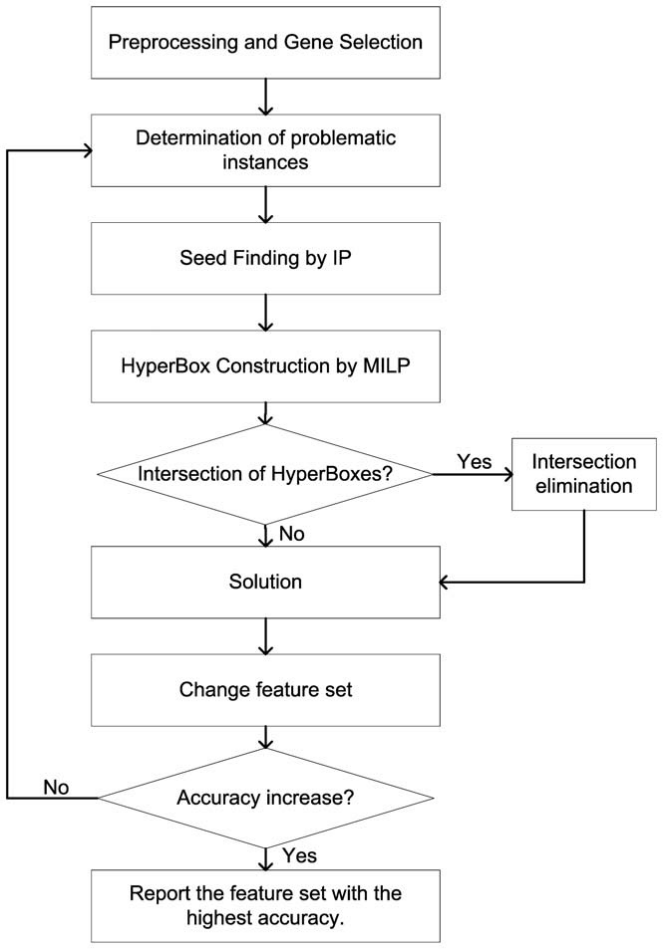
The flowchart of the algorithm.

**Figure 2:**
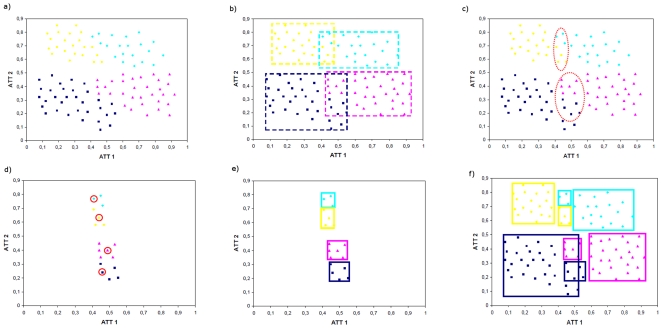
The illustrative two dimensional classification problem. a) The two-dimensional four-classes illustrative example. Each color represents one class. b) The determination of boundaries for corresponding classes for all samples. c) The determination of problematic samples. d) The identification of representative samples (seeds) from each class using pure IP. e) Construction of hyper-boxes for problematic samples using MILP. f) Construction of hyper-boxes for non-problematic samples.

Figure 1 summarize the steps of hyper-box enclosure algorithm. Also, an illustrative example explaining the HBE algorithm is given in Figure 2. In the illustrative example, the problem consists of two attributes and four classes (Figure 2a). First, the maximum and the minimum attribute values are calculated for each class (Figure 2b). Then, the boundaries of the classes are compared to check whether they overlap. If the boundaries of the classes overlap, then the samples that are enclosed by other classes are identified (Figure 2c). These samples are called as ‘problematic’ samples, since they are not separable from the samples of the other classes with a single hyper-box. In the case of having large number of ‘problematic’ samples, the same procedure is repeated to reduce the total number of such samples. In some cases, applying one or two times the same procedure do not reduce the number of problematic samples as desired; therefore we use integer programming based seed finding algorithm to reduce this computational complexity.

### Seed Finding

This step is used to improve the computational efficiency by determining representative seeds for each class (Figure 2d). Seed finding is a method that selects a representative sample (seed) for each class (tumor type) and fixes assignments of these samples to their respective classes before solving the problem. The seeds improve the computational performance of the model without changing the optimal solution. The determination of seeds is a critical task: the seeds for each class must be chosen to ensure that seeds are separated well from each other as well as being a good example of the group of samples in the same class. We develop a pure integer programming (IP) formulation to accomplish this task. Samples are represented by the parameter 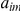 that denotes the value of attribute 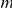 for the samples 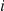. The class 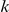 of sample 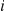 belongs to is given by the set 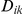. Moreover, 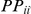 represents the distance between two samples 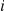 and 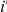. This distance is calculated using Euclidean distance in 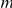 dimensional space as given in Equation (5).

As it is proven in Uney-Yuksektepe [65], the constraint set of the seed finding model has the totally unimodular property. This property theoretically guarantee that every basic feasible solution of the LP relaxation of seed finding model defined by constraint (8) is integer. Therefore, optimal solution of LP-relaxation is the optimal solution of Seed Finding model which means that solution of Seed Finding model could be easily obtained in very short time. Hence, determination of seeds is not a major undertaking due to this theoretical property. For example, the Seed Finding model is solved in 0.063 seconds for classification of leukemia. Moreover, seed finding algorithm optimally determines the corresponding seed for each class. Hence, for a given data set exactly the same instances will be selected as seeds for distinct runs of seed finding model.

Furthermore, different classification models will always develop different models (rules, trees, boxes, etc.) for different data sets. In classification problems, benchmark data sets are used in order to compare the results of different methods. As the same benchmark data sets for each tumor problem are used to compare distinct models, the comparisons are unbiased and stable for this study. For instance, the most popular data classification method Support Vector Machines (SVM) will produce different hyper-planes for perturbations in the original data set. Hence, differences between the constructed hyper-boxes of different data sets are not an interesting and problematic issue for data classification.
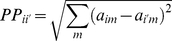
(6)

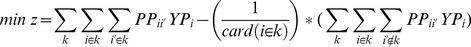
(7)

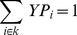
(8)


The objective of the IP-Seed problem given in Equation (6) is to minimize the distances from each seed to sample of its group (in-class distances) and maximize the average distances from each seed to the samples that belong to other classes (out-class distances). Equation (7) states that every class must have exactly one seed, and integrality of the decision variable 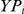 is given by 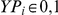.

### Construction of boxes with seeds

Training part studies are performed on a training data set composed of a number of samples 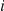. The samples are represented by the parameter aim that denotes the value of gene 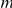 for the sample 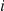. The class 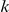 that the sample 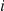 belongs to are given by the set 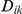. Each existing hyper-box 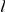 encloses a number of samples belonging to the class 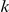. Moreover, bounds 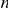 (lower, upper) of each hyper-box is determined by solving the training problem (the mathematical model is granted in Additional file 1).

Minimization of the number of misclassified samples in the data set with the minimum number of hyper-boxes is the objective of the mixed integer linear programming model. The objective function is
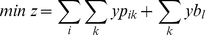
(9)where 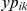 indicates the misclassification of sample 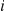 to class 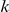 and the existence of hyper-box 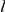 is represented by binary variable 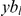.

The lower and upper bounds of the hyper-boxes are determined by the samples that are enclosed within the hyper-boxes. Hence, lower and upper bounds of hyper-boxes are calculated by related constraints. Moreover, the bounds of hyper-boxes exist if and only if this hyper-box is assigned to a class. There exist constraints that ensure the assignment of each data point to a single box and single class [66]. It is also shown in Figure 2e.

### Intersection elimination

**Figure 3:**
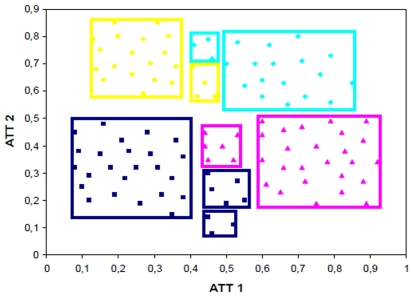
The final solution after the intersection elimination.

Since the MILP model is solved for ‘problematic samples’ only, the ‘non-problematic samples’ are assigned to hyper-boxes in a straight forward manner (Figure 2f). We define 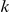 hyper-boxes for each class and assign a ‘non-problematic sample’ to corresponding newly defined hyper-box. Each ‘non-problematic sample’ is considered one by one until all of these samples are assigned to a hyper-box. Finally, the bounds of these new hyper-boxes are determined by considering the maximum and minimum attribute values of all samples in these hyper-boxes. It may be possible that the constructed hyper-boxes obtained from MILP model and defined hyper-boxes have intersections. Samples are separated from the defined hyper-box until all intersections are eliminated. The eliminated instances are grouped in a new box and intersection checking and elimination procedure is repeated until no more intersections occur between all of the constructed and defined hyper-boxes (Figure 3).

### Optimal Gene Set Finding

After the initial gene ranking, the optimal gene set is searched by using 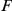 cumulative distribution function (FCDF) and the classification iteratively. FCDF is computed at each values in 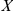 using the corresponding parameters in 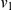 and 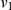. FCDF is:
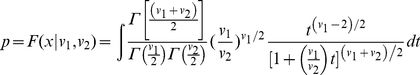
(10)where 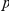 is the probability that single observation from an 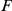 distribution with parameters 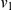 and 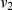. In our case, 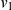 is the number of samples and 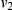 is number of samples at each class. X is the division of the variances of each gene of all samples to the variances of each gene of each sample. As a result 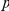 value for one class is calculated 1- FCDF. While defining the relatively irrelevant genes (the least informative genes) to leave the model within the optimal gene subset the gene with the maximum 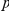 value for one of the classes is selected. In this way, the least informative gene that is indicated by very low 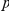 value (close to 0) for that particular class is replaced by the most informative gene that has a value (close to 1) for that particular class. As the least and most informative genes were calculated by FCDF, the least informative genes are replaced by the most informative ones, and hyper-box enclosure method is used at each iteration. The set giving the highest classification accuracy is reported as the optimal gene set. Also, the genes whose ranking scores are the highest are checked whether there is a redundancy among them by considering the pair correlation-coefficients. As a result, this approach selects the most relevant genes to the target classes and minimizes the redundancy among the selected genes to define an optimal gene set which provides the highest classification accuracy.

## Supporting Information

Supporting Information S1 MILP formulation of the hyper-box enclosure approach.
(0.05 MB PDF)

Supporting Information S2 Source codes, scripts and data files.
(0.78 MB ZIP)
