# Supplementary material for: Optimization Based Tumor Classification from Microarray Gene Expression Data
Source: PLoS One. 2011 Feb 4;6(2):e14579. doi: 10.1371/journal.pone.0014579 (PMC3033885; doi:10.1371/journal.pone.0014579)
Supplement: Supporting Information S1 — MILP formulation of the hyper-box enclosure approach. (0.05 MB PDF) [file pone.0014579.s001.pdf]

MILP Formulation of the Hyper-box Enclosure approach:

Training part studies are performed on a training data set composed of a number of samples i. The samples are represented by the parameter a_{im} that denotes the value of gene m for the sample i. The class (tumor type) k that the sample i belongs to are given by the set D_{ik}. Each existing hyper-box l encloses a number of samples belonging to the class k. Moreover, bounds n (*lower, upper)* of each hyper-box is determined by solving the training problem. M and N represents the total number of genes and bounds, respectively.

Given these parameters and the sets, the following binary and continuous variables are sufficient to model the data classification problem with hyper-boxes. The existence of hyper-box l is represented by binary variable yb_l. The binary variable ypb_{il} indicates the position (inside or outside) of the sample i with respect to box l. The binary variables ybc_{lk} and ypc_{ik} indicate the assigned class k of sample i and hyper-box l, respectively. If the sample i is within the bound n with respect to gene m of hyper-box l, then the binary variable $ypbn_{ilmn}$ is 1, otherwise 0. Similarly, $ypbm_{ilm}$ indicates whether the sample i is within the bounds of gene m of hyper-box l or not. Finally, yp_{ik} indicate the misclassification of sample i to class k. In order to define the boundaries of hyper-boxes, two continuous variables are required: X_{lmn} is the one that models bounds n for box l on gene m. Correspondingly, bounds n for box l of class k on gene m are defined with the continuous variable XD_{lkmn}.

The following MILP problem models the training part of data classification method using hyper-boxes:

$$\min z = \sum_i \sum_k yp_{ik} + \sum_k yb_l \tag{A.1}$$

subject to

$$XD_{lkmn} \leq a_{im} ypb_{il} + q(1 - ypb_{il}) \ \forall\ i,k,l,m,n|n = lower \tag{A.2}$$

$$XD_{lkmn} \leq a_{im} ypb_{il} \forall\ i,k,l,m,n|n = upper \tag{A.3}$$

$$XD_{lkmn} \leq Qybc_{ik} \ \ \forall\ k,l,m,n \tag{A.4}$$

$$\sum_k XD_{lkmn} = X_{lmn} \ \forall\ l,m,n \tag{A.5}$$

$$ypbn_{ilmn} \geq \frac{1}{Q}(X_{lmn} - a_{im}) \ \ \forall\ i,l,m,n|n = upper \tag{A.6}$$

$$ypbn_{ilmn} \geq \frac{1}{Q}(a_{im} - X_{lmn}) \;\; \forall\, i, l, m, n | n = lower \qquad \text{(A.7)}$$

$$\sum_l ypb_{il} = 1 \;\; \forall\, i \qquad \text{(A.8)}$$

$$\sum_k ypcb_{ik} = 1 \;\; \forall\, i \qquad \text{(A.9)}$$

$$\sum_l ypb_{il} = \sum_l ypc_{ik} \;\; \forall\, i \qquad \text{(A.10)}$$

$$\sum_k ypc_{lk} = \; yb_l \;\; \forall\, l \qquad \text{(A.11)}$$

$$ybc_{lk} \geq \sum_i ypb_{il} \;\; \forall\, l, k \qquad \text{(A.12)}$$

$$ybc_{lk} \leq \sum_i ypc_{ik} \;\; \forall\, l, k \qquad \text{(A.13)}$$

$$\sum_n ypbn_{ilmn} - ypbm_{ilm} \; \leq N - 1 \;\; \forall\, i, l, m \qquad \text{(A.14)}$$

$$\sum_m ypbm_{ilm} - ypc_{ik} \; \leq M - 1 \;\; \forall\, i, l, k \qquad \text{(A.15)}$$

$$ybc_{ik} \leq yp_{ik} \;\; \forall\, i, k \not\in D_{ik} \qquad \text{(A.16)}$$

$$X_{lmn}\, , XD_{lkmn} \geq 0 \qquad \text{(A.17)}$$

$$yb_l, ypb_{il}, ybc_{ik}, ybc_{lk}, ypbn_{ilmn}, ypbm_{ilm}, yp_{ik} \; \in \{0,1\} \qquad \text{(A.18)}$$

Minimization of the misclassified samples in the data set with the minimum number of hyper-boxes is the objective of the MILP model given in (A.1). The lower and upper bounds of the hyper-boxes are determined by the samples that are enclosed within the hyper-boxes. Hence, lower and upper bounds of hyper-boxes are calculated by equations (A.2) and (A.3), respectively. Eq. (A.4) enforces the bounds of hyper-boxes exist if and only if this hyper-box is assigned to a class. The relationship between two continuous variables is given in Eq. (A.5). The position of a sample with respect to the bounds on gene *m* for a hyper-box is given in Eqs. (A.6) and (A.7). The binary variable $ypbn_{ilmn}$ helps to identify whether the sample *i* is within the hyper-box *l*. Two constraints, one for the lower bound and one for the upper bound, are needed for this purpose (Eqs. (A.6) and (A.7)). Since these constraints establish a relation between continuous and binary variables, a large parameter, *Q*, is included. *Q* generally takes the maximum gene expression value in the data set. The assignment of a sample to a single hyper-box *l* and a single class *k* is established by the equations (A.8) and (A.9), respectively. The equivalence between Eqs. (A.8) and (A.9) is given in Eq. (A.10); indicating that if there is a sample in the class *k*, then there must be a hyper-box *l* to represent the class *k* and vice versa. The existence of a hyper-box implies the assignment of that hyper-box to a class as

shown in Eq. (A.11). If a class is represented by a hyper-box, there must be at least one sample within that hyperbox as in Eq. (A.12). In the same manner, if a hyper-box represents a class, there must be at least a sample within that class as given in Eq. (A.13). The Eq. (A.14) represents the condition of a sample being within the bounds of a box in gene m. If a sample is within the bounds of all genes of a box, then it must be in the box as shown in Eq. (A.15). When a sample is assigned to a class that it is not a member of, a penalty applies as indicated in Eq. (A.16). Finally, last two constraints Eq. (A.17) and (A.18) give nonnegativity and integrality of decision variables. The model has $lmn + lkmn$ continuous variables, $l + lk + 3ik + il + ilmn + ilm$ binary variables and $O(iklm)$ constraints.
