# Supplementary material for: Optimization Based Tumor Classification from Microarray Gene Expression Data
Source: PLoS One. 2011 Feb 4;6(2):e14579. doi: 10.1371/journal.pone.0014579 (PMC3033885; doi:10.1371/journal.pone.0014579)
Supplement: Supporting Information S2 — Source codes, scripts and data files. (0.78 MB ZIP) [file pone.0014579.s002.zip › S2/Readme.docx]

Before the run, make sure that you have GAMS&MATLAB interface.To install the interface please

read “doc.pdf” in the “matgams.zip” file. Also, GAMS and CPLEX licences are needed to run the code.

**To run the code:**

Open “driver_nfold.m” for n-fold-cross-validation or “driver_testset.m” for test-set. Then just click on

“run”. The percentage accuracy will be on the command window of MATLAB.

In driver_nfold.m you can change the fold by changing the variable “fold=10”. On test set, you

should enter the number of samples in training and test set. Please delete the generated files after

you run “driver_testset.m”.

This matlab(“driver_nfold.m” or “driver_testset.m”) file takes the “input.xls” as input and prepare

input files of GAMS scripts, and then calls gams scripts.

Input format should be the same as it is in “input.xls”.

Please look at “TestResult_New.txt” for more detailed results such as predicted classes of each

sample and corresponding boxes.

There are 16 intersection elimination scripts(all are same);however you dont need to use all of them.

The number of intersection elimination scripts that you should use depend on the complexity of the

data. If you dont change anything, the code will use 16 of them, though some may be unnecessary.

However the result wont change.
