# Supplementary material for: Optimization Based Tumor Classification from Microarray Gene Expression Data
Source: PLoS One. 2011 Feb 4;6(2):e14579. doi: 10.1371/journal.pone.0014579 (PMC3033885; doi:10.1371/journal.pone.0014579)
Supplement: Supporting Information S2 — Source codes, scripts and data files. (0.78 MB ZIP) [file pone.0014579.s002.zip › S2/matgams.zip!/doc.pdf]

MATLAB and GAMS: Interfacing Optimization and Visualization Software *

Michael C. Ferris†

May 26, 2005

Abstract

This document briefly describes a link between GAMS and MATLAB, both of which the user is assumed to have already. The software gives MATLAB users the ability to use all the optimization capabilities of GAMS, and allows visualization of GAMS models directly within MATLAB. The most recent version can be downloaded directly from `http://www.cs.wisc.edu/math-prog/matlab.html`

1 Introduction

Optimization is becoming more widely used in many application areas as can be evidenced by its appearance in software packages such as Excel and MATLAB. While the optimization tools in these packages are useful for small-scale nonlinear models (and to some extent for large linear models), the lack the ability to perform automatic derivatives makes them impractical for large scale nonlinear optimization. In sharp contrast, modeling languages such as GAMS and AMPL have had such a capability for many years, and have been used in many practical large scale nonlinear applications.

On the other hand, while modeling languages have some capabilities for data manipulation and visualization (e.g. Rutherford's GNUPLOT), to a large extent specialized software tools like Excel and MATLAB are much better at these tasks.

This paper describes a link between GAMS and MATLAB. The aim of this link is two-fold. Firstly, it is intended to provide MATLAB users with a sophisticated nonlinear optimization capability. Secondly, the visualization tools of MATLAB are made available to a GAMS modeler in a easy and extendable manner so that optimization results can be viewed using any of the wide variety of plots and imaging capabilities that exist in MATLAB.

*This material is based on research supported by the Air Force Office of Scientific Research Grant F49620-98-1-0417, the National Science Foundation Grant CCR-9619765 and GAMS Corporation.

†Computer Sciences Department, University of Wisconsin – Madison, 1210 West Dayton Street, Madison, Wisconsin 53706 (`ferris@cs.wisc.edu`)

We first give a simple example of a nonlinear optimization problem that would benefit from this capability and describe the steps that are needed in order to use our interface in this application.

2 Installation

The next two subsections describe the installation procedure for a PC and a UNIX workstation respectively.

2.1 PC

First of all, you need to install both MATLAB and GAMS on your machine. We will assume that the relevant system (installation) directories are

`c:\matlab and c:\gams`

Next, you need to copy the file matout.gms into the GAMS library directory.

```
C:\>  copy matout.gms c:\gams\inclib\matout.gms
```

If you do not have write permission in the GAMS library directory, then you can put this file into whatever directory you run the MATLAB m-file from and replace any "libinclude" below with "batinclude". Alternatively, you can use the "idir" command line argument to GAMS to change the library directory "per job" to a directory where you have write permission.

You need to put the interface file into a directory on your MATLAB path. To do this, you can either update your MATLAB path from within MATLAB to include the directory where you have stored the "gams.dll" file, or put this executable onto the standard MATLAB path. For example,

```
C:\>  copy gams.dll c:\matlab\toolbox\local\gams.dll
```

since

`matlab\toolbox\local`

directory is automatically on your MATLAB path.

The third step is to ensure that the GAMS system directory is on your normal (windows) path.

- On an XP machine, you should set the environment variable `PATH` from the Control Panel to include

  `c:\gams`

  or wherever your gams system directory is. For GAMSIDE users, this may be

  `c:\Program Files\GAMS21.7`

- On Win95 or Win98, you should edit the "autoexec.bat" file (probably located on your c: drive and add the line

  ```
  set path = %path%;c:\gams
  ```

  For safety, you should probably reboot the machine at this stage on these latter platforms.

At this stage, the installation should be complete.
To test the installation, carry out the following steps.

1. Start up matlab.
2. In the matlab command window, change directories to the examples directory provided as part of the distribution. (This directory contains at least two files, testinst.m and testinst.gms that are required for this test.)
3. Run the example "testinst" that is found in the examples directory of the distribution. At the MATLAB prompt you just type:

   ```
   >> testinst
   ```

   The resulting output will depend on the platform on which you run this from. It should include the output given below.

```
Q =

    name: 'Q'
     val: [3x3 double]


ans =

     1     0     0
     0     1     0
     0     0     1


Q =

     2     0     0
     0     2     0
     0     0     2


J =

    '1'
```

```
    '2'
    '3'

Q = 

    name: 'Q'
     val: [3x3 double]


J = 

    name: 'J'
     val: {3x1 cell}

Q = 

    name: 'Q'
     val: [3x3 double]


J = 

    name: 'J'
     val: {3x1 cell}

A = 

    name: 'A'
     val: [2x3 double]


ans =

     3     0     0
     0     3     0
     0     0     3


ans = 

    '1'
    '2'
    '3'


ans =
```

```
0     2    -5
2     0     2
```

If you get the error "failed to spawn process" then you have most likely not set up your system path to include the GAMS system directory. Go back to the third step above.

2.2 UNIX

First of all, you need to install both MATLAB and GAMS on your machine. We will assume that the relevant system (installation) directories are

`/usr/local/matlab and /usr/local/gams`

Next, you need to copy the file matout.gms into the GAMS library directory.

```
%     cp matout.gms /usr/local/gams/inclib/matout.gms
```

If you do not have write permission in the GAMS library directory, then you can put this file into whatever directory you run the MATLAB m-file from and replace any "libinclude" below with "batinclude". Alternatively, you can use the "idir" command line argument to GAMS to change the library directory "per job" to a directory where you have write permission.

You need to put the interface file into a directory on your MATLAB path. To do this, you can either update your MATLAB path from within MATLAB to include the directory where you have stored the "gams.***" file, or put this executable onto the standard MATLAB path. For example, on a Solaris UNIX system,

```
%  cp gams.mexsol ~/matlab/gams.mexsol
```

On a linux system,

```
%  cp gams.mexglx ~/matlab/gams.mexglx
```

Note that the `/matlab` directory (that you can create) is automatically on your MATLAB path. While there may be other mex files in the directory, we currently only maintain the PC, Solaris and Linux versions of these codes.

The GAMS system directory should also be in your normal path; this can be effected using:

```
%     set path = (/usr/local/gams $path)
```

At this stage, the installation should be complete.

To test the installation, carry out the following steps.

1. Start up matlab.
2. In the matlab command window, change directories to the examples directory provided as part of the distribution. (This directory contains at least two files, testinst.m and testinst.gms that are required for this test.)

3. Run the example “testinst” that is found in the examples directory of the distribution. At the MATLAB prompt you just type:

```
>> testinst
```

   The resulting output will depend on the platform on which you run this from. It should include the output given in the previous subsection.

3 Simple use: returning values

The first step is to generate a working GAMS model. For example, we can set up a simple model file to solve a quadratic program

$$\begin{array}{ll} \min_x & \frac{1}{2}x^T Q x + c^T x \\ \text{subject to} & Ax \geq b, x \geq 0 \end{array}$$

This file is as follows:

```
set i /1*2/,
    j /1*3/;
alias (j1,j);

parameter
Q(j,j1) /
        1 .1 1.0
        2 .2 1.0
        3 .3 1.0 /,
A(i,j) /
        1 .1 1.0
        1 .2 1.0
        1 .3 1.0
        2 .1 -1.0
        2 .3 1.0 /,
b(i) /
        1 1.0
        2 1.0 /
c(j) /
        1 2.0 /;

variable obj;
positive variable x(j);

equation cost, dual(i);

cost.. obj =e=
  0.5*sum(j,x(j)*sum(j1,Q(j,j1)*x(j1))) + sum(j,c(j)*x(j));
```

```
dual(i)..   sum(j, A(i,j)*x(j)) =g= b(i);

model qp /cost,dual/;

solve qp using nlp minimizing obj;
```

This model will run directly at the command prompt using

```
gams qp
```

The optimal value is 0.5. If you use the example file provided with the distribution, you should make sure that no files named "matglobs.gms" and "matdata.gms" currently exist.

In order to run the same model within MATLAB and return the solution vector x back into the MATLAB workspace, one change is required to the GAMS file, namely to add the line

```
$libinclude matout x.l J
```

after the solve statement. This just writes out the level values of the solution to a file that can be read back into MATLAB. In MATLAB, you just execute the following statement:

```
>>  x = gams('qp');
```

This command just executes "gams qp"using a system call, and then collects the results, returning them back to MATLAB as a structure with two fields, a "name" and a "val". In this example name would be set to "x.l" and val would be a 3x1 vector of doubles, holding the optimal solution values. If you prefer just to get a MATLAB vector containing the optimal values, you can change the output style from GAMS using

```
>>  gams_output = 'std';
>>  x = gams('qp');
```

In this case, x will just be a 3 by 1 vector containing the solution values $(0, 0, 1)$, and the name "x.l" will be discarded. Note that this example is given as part of the examples directory of the distribution, see `do_qp.m`.

If you have an m-file that states `gams('foo')`, then it is important to note that the file foo.gms must be found in the directory that matlab executes the command "gams('foo')".

If you wish to retrieve multiple vectors (matrices) from the solve, then other return values can be written out from the GAMS file. For example, if you want the solution vector and the multipliers, then add the two lines

```
$libinclude matout x.l J
$libinclude matout dual.m I
```

to your GAMS file and issue the following MATLAB command:

```
>>  [x,u] = gams('qp');
```

Note that order is important here. The first output (x.l) from GAMS is put into the first output argument of the MATLAB call, x. The multipliers are the second argument from GAMS, and this gets returned as the "u" vector to MATLAB. If the numbers of outputs in these files do not match, the system defaults appropriately (it discards GAMS output, or does not assign to the MATLAB return arguments). Multiply dimensioned parameters can be returned by appending the appropriate indexing sets after I or J in the above.

One piece of information that may be needed within MATLAB is the modelstat and solvestat values generated by GAMS for the solves that it performed. This is easy to generate, and is given as the example `do_status.m`. This example is generated by taking the standard gamslib trnsport example, and adding the following lines to the end:

```
set stat /modelstat,solvestat/;
parameter returnStat(stat);
returnStat('modelstat') = transport.modelstat;
returnStat('solvestat') = transport.solvestat;
$libinclude matout returnStat stat
```

Note that the relevant status numbers are stored in GAMS into the parameter returnStat that is returned into MATLAB using the standard "matout" mechanism outlined above. Once these are returned to MATLAB they can be printed out or manipulated as follows:

```
gams_output = 'std';
s = gams('trnsport');
fprintf('modelstat = %d\n',s(1));
fprintf('solvestat = %d\n',s(2));
```

Further examples of the use of this utility are given in the ensuing sections. In particular, it is possible to return strings from GAMS into MATLAB, and to pass back cell arrays containing the text labels of (ordered) GAMS sets. However, we will first look at the issue of updating parameter values in the GAMS file directly from MATLAB.

4 Simple use: modifying parameters

It is often useful to run a series of optimizations, at each iteration changing just a small number of parameters. For example, one may wish to try multiple starting points or solve multiple quadratic programs, each of which has a different right hand side. This can be carried out easily with the GAMS interface by setting up multiple inputs to the GAMS file. **Note that the mechanism to do this has changed in Matlab version 7.0** (due to the removal of a critical mex interface function).

For example, if you wish to update the vector b, then the relevant call in MATLAB would be:

```
>>  b = [1; 2];
>>  [x,u] = gams('qp','b')
```

Note that in order to pass the vector "b" into gams, you need to use a string argument so that gams can extract both the name "b", and the data that is stored in the vector b. This call writes out the new values of "b" to a file that we need to include into the model. Thus, we also need to update the "qp.gms" file to read in the new values. We suggest that the following line is added immediately before the solve statement of "qp.gms":

```
$if exist matdata.gms $include matdata.gms
```

Thus, if the file "matdata.gms" does not exist, the original data is used. Otherwise, the values of "b" are killed at GAMS compile time and replaced by their new values of 1 and 2 respectively. Note that this does not merge the new values of b with the old values, but kills off the existing values and puts in the new values given as b. The GAMS user must be aware of the fact that the existing values are replaced (as data) at compile time. If the GAMS program contains any execution time statements modifying the parameter (for example, assignment statements), these modifications will continue to overwrite the data that is imported from MATLAB.

More sophisticated GAMS users may wish to update "global" variables (strings) in the GAMS model from MATLAB. This can be carried out by passing a MATLAB "string" as an input argument to the gams call. The interface takes this string and writes it into a file "matglobs.gms" using a `$setglobal` command.

It is also possible to modify GAMS set declarations from MATLAB. The technique to carry this out is to declare a MATLAB "logical" array, and pass this on the command line to GAMS. For example, if the file `setset.gms` contains the following lines:

```
set y(*,*);
$if exist matdata.gms $include matdata.gms
display y;
```

then

```
>>  y = ([1 0 2; 2 0 1] > 0);
>>  gams('setset','y');
```

ensures that $Y = \{(1,1),(1,3),(2,1),(2,3)\}$.

To pass on set label strings (instead of indices) from MATLAB to GAMS, see a later section.

In summary, the three ways you may wish to update the data in your GAMS file are:

1. If you wish to set or update the values of global strings in the GAMS file, then add the following line after any relevant `$setglobal` commands in the GAMS file;

```
$if exist matglobs.gms $include matglobs.gms
```

2. To update any scalars, parameters or sets, add the following line after the parameter/set has been declared in the GAMS file:

   ```
   $if exist matdata.gms $include matdata.gms
   ```

3. To retrieve values from GAMS in MATLAB, use the GAMS batch utility "matout" as follows:

   ```
   $libinclude matout v.l I J
   $libinclude matout lb I J
   ```

4. The next example above shows how strings can be sent back to MATLAB. The corresponding MATLAB variable will be set to the string 'grunt'.

   ```
   $libinclude matout matstr 'grunt'
   ```

5. In some GAMS programs, strings are found as labels in system variables or as fields of some of the declared parameters, sets, variables or equations. The following example shows how to retrieve these strings back into MATLAB.

   ```
   $libinclude matout matlbl system.title
   $libinclude matout matlbl v.ts
   ```

   In the first example, the string that is set in the $title command is sent back as a MATLAB string. In the second example, the symbol text associated with the variable v is passed back into a MATLAB string.

6. The final example shows how to pass back labels associated with set elements. These come in two ways, either as text labels (tl) or as text elements (te).

   ```
   $libinclude matout J
   $libinclude matout J tl
   $libinclude matout J te
   ```

   The first two matout statements return the text labels associated with the GAMS set J. The third example passes back the text elements (the strings associated with each of the elements of the set). The return arguments in MATLAB are cell arrays, each element of which is a string corresponding to the element of the GAMS set. This is a useful mechanism to retrieve the GAMS labels within the MATLAB envionment.

Note that we can only setglobals on input or change the values of parameters. It might be convenient to update level values, etc on input but this is currently possible only via a parameter.

To change the output parameters from structures to (standard MATLAB) matrices the variable

```
  gams_output = 'std';
```

must be set in the m-file that calls "gams".

It is possible to call the output routine "matout" within a GAMS loop. However, the "matout" procedure must first be called outside of the loop to initialize the output file. Thus the following example would produce 11 outputs, one for each run through the loop.

```
scalar x /-1/;
set iter /i1*i10/;

$libinclude matout iter

loop(iter,
  x = ord(iter);
$libinclude matout x
);
```

Figures 1 and 2 show how to use this interface to implement a replacement "qpopt" for the Optimization Toolbox routine "qp", assuming that you have an NLP solver for GAMS. The implementation described in the two files "qpopt.m" and "qpopt.gms" uses the same calling sequence but carries out the optimization using whatever nlp solver is in use by the GAMS system.

Another point to note for efficiency is that MATLAB sparse matrices are written out to the matdata.gms file ordered by rows. This is carried out in the interface by transposing the sparse matrix internally, and then writing out the appropriate values in row oriented format. This greatly increases the speed of reading in large sparse matrices into GAMS. Dense matrices are not treated in this manner, so such matrices may read into GAMS more slowly.

5 Advanced Use: Labels

We have briefly mentioned the fact that set labels can be returned from the GAMS file to MATLAB. We now describe in more detail the MATLAB syntax that works with these labels. In this context, both structures and cells are used in the MATLAB environment.

The basic object that is used in the MATLAB interface is a structure. In all the calls we have mentioned so far, the structure has at least two fields, namely a "name" field that holds the GAMS name of the object, and a "val" field that is used for the values that are passed between GAMS and MATLAB. (If the argument passed to or from MATLAB is not a structure, then the argument

```
function [X,lambda]=qpopt(H,f,A,B,vlb,vub,X0,neq)

% Handle missing arguments
   if nargin < 8, neq = 0;
      if nargin < 7, X0 = [];
         if nargin < 6, vub = [];
           if nargin < 5, vlb = [];
end, end, end, end
[ncstr,nvars]=size(A);
nvars = max([length(f),length(H),nvars]); % In case A is empty

if isempty(neq), neq = 0; end
if isempty(X0), X0=zeros(nvars,1); end
if isempty(vlb), vlb=-inf*ones(nvars,1); end
if isempty(vub), vub=inf*ones(nvars,1); end
if isempty(A), A=zeros(0,nvars); end
if isempty(B), B=zeros(0,1); end

% Expect vectors
f=f(:); B=B(:); X0 = X0(:);
vlb = vlb(:); vub = vub(:);

if  norm(H,'inf')==0 | isempty(H)
  H=[];
else
  % Make sure it is symmetric
  if norm(H-H','inf') > eps
    H = (H+H')*0.5;
  end
end

% fool interface to make A a matrix, b a vector
if ncstr == 1
  A(ncstr+1,1) = 0;
  B(ncstr+1) = 0;
end
neq = int2str(neq);
ncstr = int2str(ncstr);
nvars = int2str(nvars);
gams_output = 'std';
[X,lambda] = gams('qpopt','H','f','A','B','vlb','vub','X0','neq','ncstr','nvars');
```

Figure 1: Example: qpopt.m

```
$onempty
$include matglobs.gms

$if %ncstr% == 0 $goto unconstr
set i /1*%ncstr%/, ieq(i), ilt(i), j /1*%nvars%/;

ieq(i) = yes$(ord(i) le %neqcstr%);
ilt(i) = not ieq(i);
$goto probdef

$label unconstr
set i / dummy /, ieq(i) //, ilt(i) //, j /1*%nvars%/;

$label probdef
parameter H(j,j) //, f(j) //, A(i,j) //, b(i) //,
          vlb(j) //, vub(j) //, x0(j) //;

$include matdata.gms

alias(j1,j);

variables x(j), obj;
equations cost, equality(i), leineq(i);

cost..  obj =e= 0.5*sum(j,x(j)*sum(j1,H(j,j1)*x(j1)))
             + sum(j,f(j)*x(j));

equality(ieq)..  sum(j,A(ieq,j)*x(j)) =e= b(ieq);

leineq(ilt)..  sum(j,A(ilt,j)*x(j)) =l= b(ilt);

x.lo(j) = vlb(j); x.up(j) = vub(j); x.l(j) = x0(j);

model qp /all/;
solve qp using nlp minimizing obj;

parameter lambda(i);
lambda(ieq) = equality.m(ieq); lambda(ilt) = leineq.m(ilt);

$libinclude matout x.l j
$libinclude matout lambda i
```

Figure 2: Example: qpopt.gms

is used as the val field, and the name defaults to the MATLAB name of the argument.) A third optional field of the structure is the "labels" field. If this field is used, it should be a cell array. The size of this array should be one by the number of dimensions of the val field. Each entry in the cell array should be another cell array, this array being one by the dimension of the corresponding dimension of the val field. This second cell array should hold the strings that will be used as labels for the val parameter.

For example, if we have a 2x2 matrix with the following labeling

	fred	bill
pizza	1	0
beer	2	3

the MATLAB code to send this to GAMS would be:

```
>> E.name = 'likes';
>> E.val = [1 0; 2 3];
>> E.labels = {{'pizza' 'beer'} {'fred' 'bill'}};
>> gams('labels',E)
```

Here the GAMS file "labels.gms" is simply:

```
set people / fred, bill /,
    commod / pizza, beer /;

parameter likes(commod,people);
$if exist matdata.gms $include matdata.gms

display likes;
```

A simple extension of the above allows one to declare the set labels within GAMS and pass them back to MATLAB automatically. The key idea is to use a global variable in the GAMS code, that gets set differently from MATLAB during two calls to GAMS. In the first call, we just return the values of the set labels, while in the second call, these labels are used to assign values to the parameters. The resulting MATLAB and GAMS code is shown below:

```
>> getsets = 'yes';
>> [commod,people] = gams('labels','getsets')
>> E.name = 'likes';
>> E.val = [1 0; 2 3];
>> E.labels = {commod.val people.val};
>> gams('labels',E)
```

Here the GAMS file "labels.gms" is simply:

```
$if exist matglobs.gms $include matglobs.gms

set people / fred, bill /,
    commod / pizza, beer /;
```

```
$if not setglobal getsets $goto contexec
$libinclude matout commod
$libinclude matout people
$exit

$label contexec

parameter likes(commod,people);
$if exist matdata.gms $include matdata.gms

display likes;
```

A simplification to the MATLAB calling sequence is available for vectors. For single dimensioned val fields, the labels field can just be a cell array of strings (as opposed to a 1x1 cell array containing a cell array of strings). For example, we could use the following MATLAB commands:

```
>> F.name = 'vector';
>> F.val = [1 0];
>> F.labels = commod.val;
>> gams('vector',F)
```

Note that if labels are used, then a label must be provided for each dimension of the val field. If the default indexing '1','2',..,'m' is suitable for a particular dimension of the parameter, then the corresponding MATLAB commands produce the required cell for that dimension

```
cellstr(num2str([1:m]'))
```

Note that the MATLAB variable 'm' here needs to be an integer. Future implementations may allow an empty cell to be passed here instead.

As an alternative, the set labels can be generated in MATLAB and passed through to GAMS. To create the sets `people` and `commod` from MATLAB, the following MATLAB sequence can be used:

```
>> P.name = 'people';
>> P.labels = {'fred' 'bill'};
>> P.val = logical(ones(size(P.labels)));
>> C.name = 'commod';
>> C.labels = {'pizza' 'beer'};
>> C.val = (ones(size(C.labels)) > 0);
>> E.name = 'likes';
>> E.val = [1 0; 2 3];
>> E.labels = {C.labels P.labels};
>> gams('labels2',P,C,E)
```

Note that the third and sixth lines set up the respective val fields as logical arrays in MATLAB. The GAMS file "labels2.gms" is now simply:

```
set people, commod;

parameter likes(commod,people);
$if exist matdata.gms $include matdata.gms

display likes;
```

6 Changing default behaviour

There are currently four variables that can be set in the MATLAB program that calls GAMS to affect the behaviour of the program. These are as follows.

- `gams_output` If this is set to the string 'std', then the output from the GAMS call will be returned as a string or matrix. Otherwise, the default is to return a MATLAB structure with two fields, a "name" field containing the GAMS name, and a "val" field that contains the string or matrix.

- `gams_input` By default, the interface updates data at compile time. Thus, if execution time updates are made to the parameters before the line "$include matdata.gms" these may override the data that is provided in "matdata.gms" (i.e. from the command line). This may not be desirable. If you wish to perform execution time updates to the data, you should set `gams_input` to 'exec'. An example is given in `do_exec.m`. To understand this example, the reader should inspect the exec.lst file at each pause statement to see the effects of the different options.

- `gams_write_data` If this is set to "no" or "No", then all parameters on the call to GAMS are ignored, except the program name. This is useful for dealing with large datasets. Consider the following invocation:

  ```
  x = gams('largedata','A');
  y = gams('resolve','A');
  ```

  The first call generates a file "matdata.gms" containing the elements of the matrix A for use in the largedata.gms program. The second call rewrites a new "matdata.gms" file that again contains A. If we wish to save writing out A the second time we can use the following invocation:

  ```
  x = gams('largedata','A');
  gams_write_data = 'no';
  y = gams('resolve','A');
  clear gams_write_data;
  ```

  or the equivalent invocation:

  ```
  x = gams('largedata','A');
  ```

```
gams_write_data = 'no';
y = gams('resolve');
clear gams_write_data;
```

- gams_show This is only relevant on a Windows platform. This controls how the "command box" that runs GAMS appears on the desktop. The three possible values are:
  - 'minimized' (default): The command prompt appears iconified on the taskbar.
  - 'invisible' : No command prompt is seen.
  - 'normal' : The command prompt appears on the desktop and focus is shifted to this box.

7 Advanced Use: Plotting

One of the key features of the GAMS/MATLAB interface is the ability to visualize optimization results obtained via GAMS within MATLAB.

Some simple examples are contained with the program distribution. For example, a simple two dimensional plot with four lines can be carried out as follows. First create the data in GAMS and export it using the matout utility outlined above:

```
$title  Examples for plotting routines via MATLAB

set t /1990*2030/, j /a,b,c,d/;

parameter a(t,j);
a("1990",j) = 1;
loop(t,  a(t+1,j) = a(t,j) * (1 + 0.04 * uniform(0.2,1.8)); );

parameter year(*); year(t) = 1989 + ord(t);

* Omit some data in the middle of the graph:

a(t,j)$((year(t) gt 1995)*(year(t) le 2002)) = NA;

$libinclude matout a t j
$libinclude matout t te
$libinclude matout j
$libinclude matout matlbl system.title
```

We make an assumption that the user will write the plotting routines in the MATLAB environment. To create the plot in MATLAB, the sequence of MATLAB commands in Figure 3 should be input (saved as do_plot.m):

```
gams_output = 'std';
[a,xlabels,legendset,titlestr] = gams('simple');
figure(1)

% Plot out the four lines contained in a
% Format using the third argument
plot(a,'.-');

% only put labels on x axis at 5 year intervals
xtick = 1:5:length(xlabels);
xlabels = xlabels(xtick);
set(gca,'XTick',xtick);
set(gca,'XTickLabel',xlabels);

% Add title, labels to axes
title(titlestr)
xlabel('Year -- time step annual');
ylabel('Value');

% Add a legend, letting MATLAB choose positioning
legend(char(legendset),0);

% match axes to data, add grid lines to plot
axis tight
grid
```

Figure 3: Simple plot in MATLAB

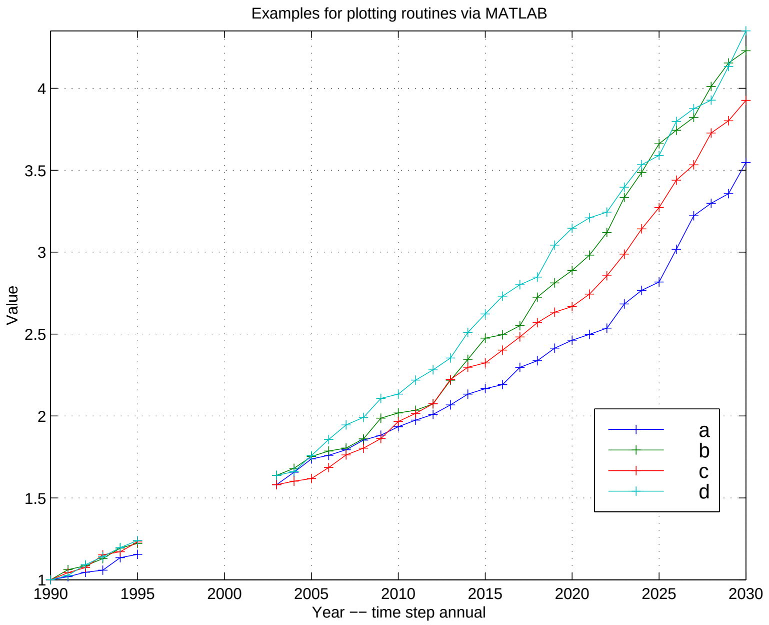

Figure 4: Simple figure created using interface

Figure 4 is an example created using this utility (and print -depsc2 simple).

MATLAB supports extensive hard copy output or formats to transfer data to another application. For example, the clipboard can be used to transfer meta files in the PC enviroment, or encapsulated postscript files can be generated. The `help print` command in MATLAB details the possibilities on the current computing platform.

Scaling of pictures is also most effectively carried out in the MATLAB environment. An example of rescaling printed out is given in Figure 5.

Note that the output of this routine is saved as a jpeg file "rescale.jpg".

Other examples of uses of the utility outlined in this paper can be found in the "m" files:

```
do_ehl
do_obstacle
taxplot
plotit
plotngon
```

Acknowledgements

The author would like to thank Steven Dirkse, Todd Munson and Thomas Rutherford for constructive comments on the design and improvement of this

```
do_plot;
fpunits = get(gcf,'PaperUnits');

set(gcf,'PaperUnits','inches');
figpos = get(gcf,'Position');
pappos = get(gcf,'PaperPosition');
newpappos(1) = 0.25;
newpappos(2) = 0.25;
newpappos(3) = 4.0;
% get the aspect ratio the same on the print out
newpappos(4) = newpappos(3)*figpos(4)/figpos(3);

set(gcf,'PaperPosition',newpappos),
print -djpeg100 rescale.jpg
set(gcf,'PaperPosition',pappos);
set(gcf,'PaperUnits',fpunits);
```

Figure 5: Rescaling printed output from MATLAB

tool.
